# Supplementary material for: Intra-Specific Variation in Desiccation Tolerance of Citrus sinensis ‘bingtangcheng’ (L.) Seeds under Different Environmental Conditions in China
Source: Int J Mol Sci. 2023 Apr 17;24(8):7393. doi: 10.3390/ijms24087393 (PMC10139128; doi:10.3390/ijms24087393)
Supplement: Supplementary file 1 [file ijms-24-07393-s001.zip › Supplemental Figures.pdf]

Supplemental Figures

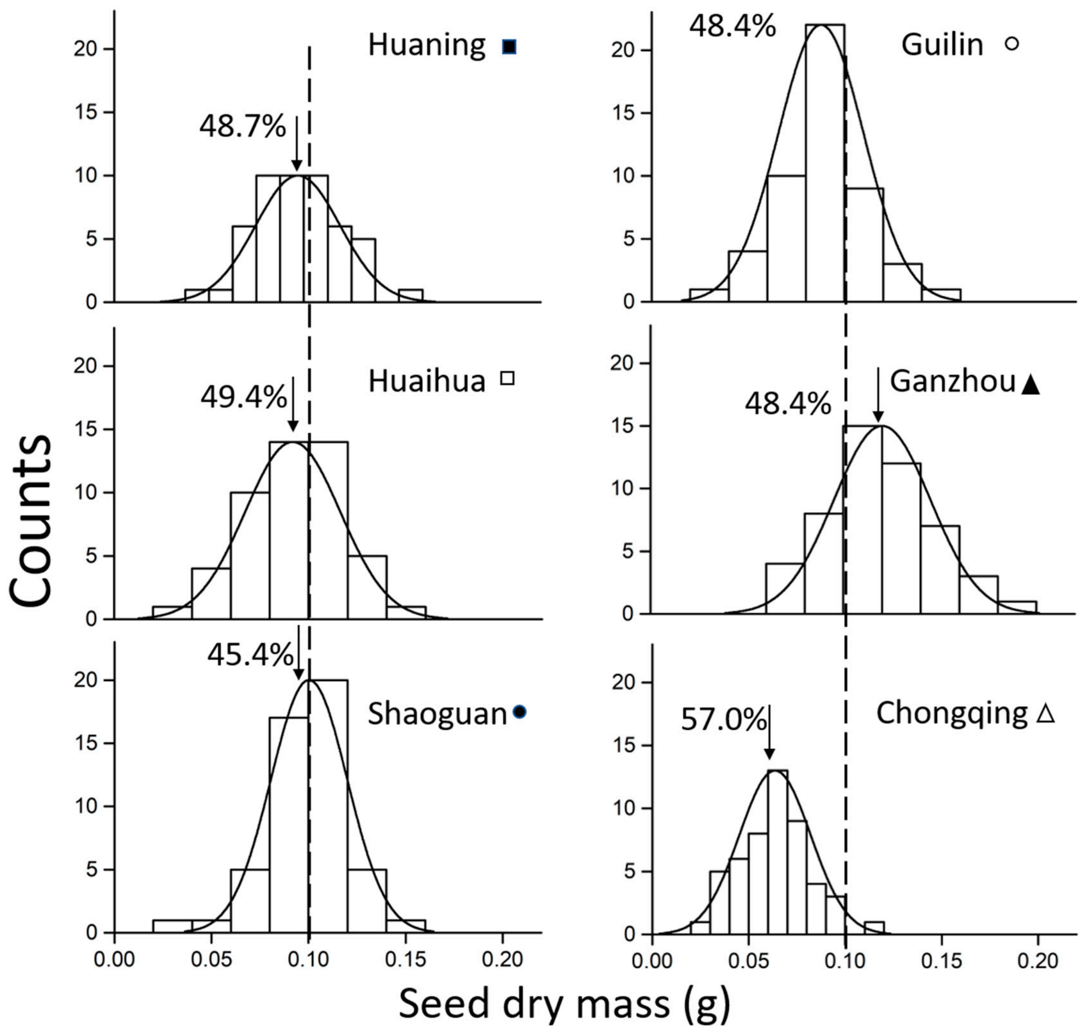

Figure S1. The distribution of 50 individual seed dry weights for six different seed lot of *C. sinensis* from Huaning (■), Huaihua (□), Shaoguan (●), Guilin (○), Ganzhou (▲), Chongqing (△). The arrows on the figure correspond to the mean water contents of each seed lot with fresh seeds.

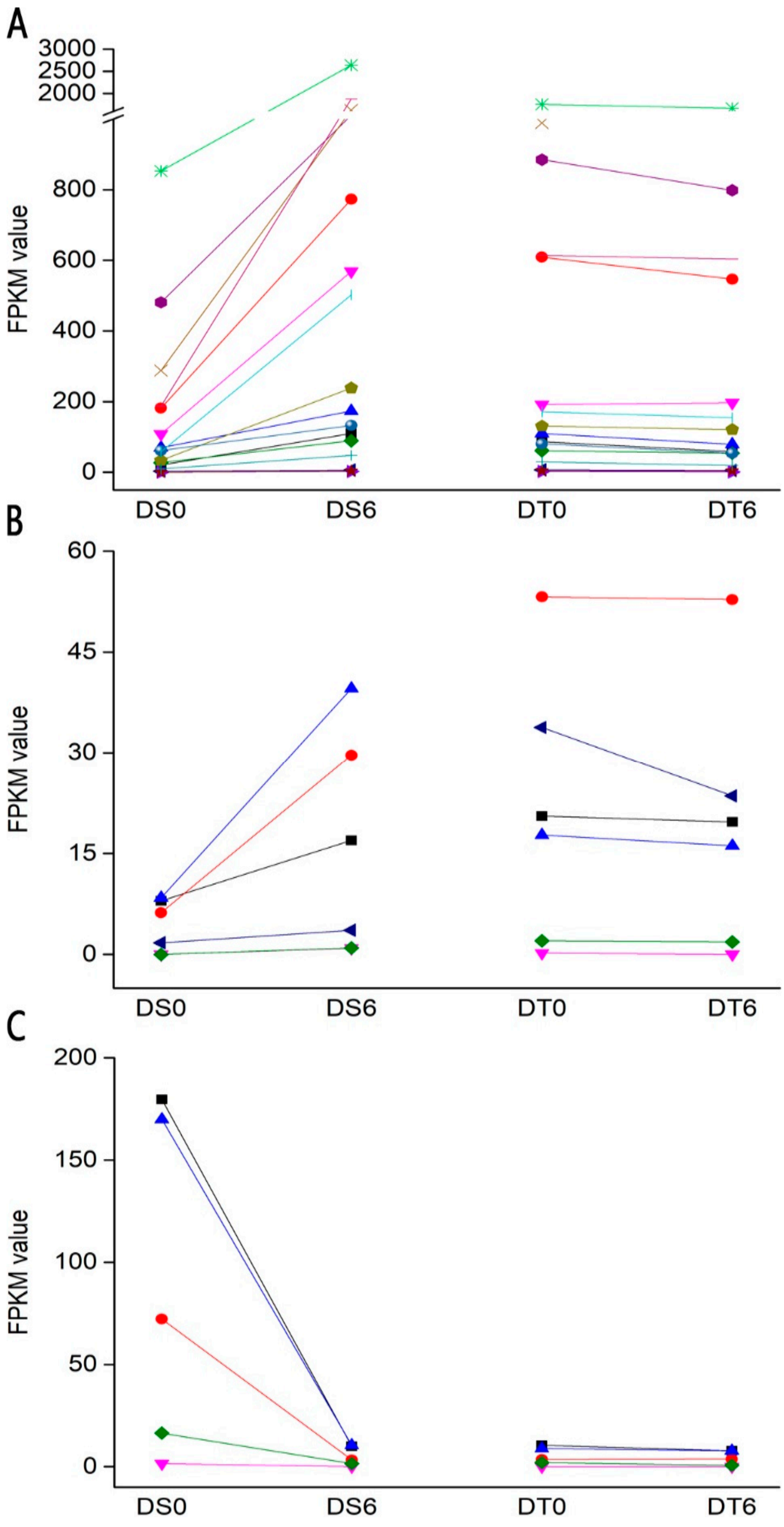

Figure S2. Analysis of key gene families that were differentially expressed (DEGs: FDR < 0.05 and absolute log2 fold change ≥ 1) in DS seeds following drying. (**A**) Heat shock protein (HSP) family genes, (**B**) peroxidase genes, and (**C**) cytochrome P450 genes. DS0: desiccation sensitive seeds desiccated for 0 d, DS6: desiccation sensitive seeds desiccated for 6 d, DT0: desiccation tolerant seeds desiccated for 0 d, DT6: desiccation tolerant seeds desiccated for 6d.

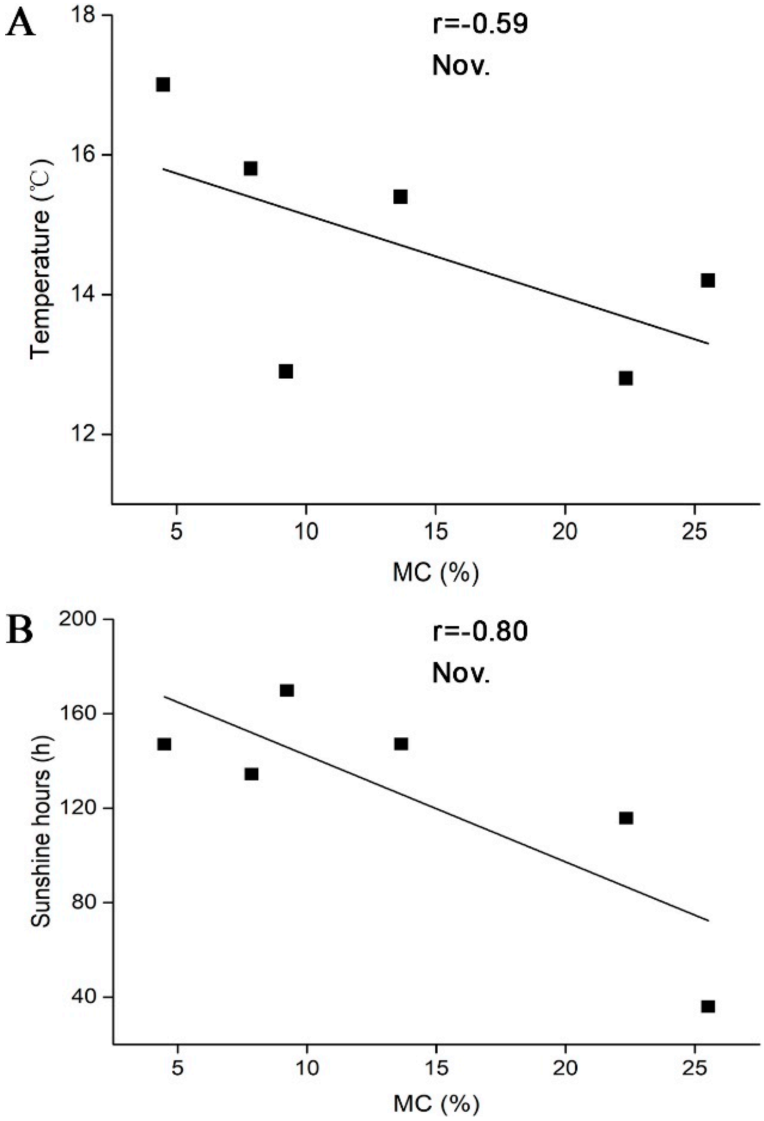

Figure S3. Correlation between the seed MC at which germination was interpolated to be probit 5.5 and *Citrus sinensis 'bingtangcheng'* seed lot environmental conditions of the collecting sites. (**A**) temperature (°C), (**B**) sunshine hours (h). Climate conditions were the temperature of November (the last month before fruit harvesting).
