# Supplementary material for: Intra-Specific Variation in Desiccation Tolerance of Citrus sinensis ‘bingtangcheng’ (L.) Seeds under Different Environmental Conditions in China
Source: Int J Mol Sci. 2023 Apr 17;24(8):7393. doi: 10.3390/ijms24087393 (PMC10139128; doi:10.3390/ijms24087393)
Supplement: Supplementary file 1 [file ijms-24-07393-s001.zip › Supplemental Tables.pdf]

Supplemental Tables

Table S1. Average temperature (°C) ofCitrus sinensis collection site from January to December that recorded from 1981 to 2010 for Huaning, Huaihua, Shaoguan, Guilin, Ganzhou and Chongqing.

Month	Place					
	Huaning (°C)	Huaihua (°C)	Shaoguan (°C)	Guilin (°C)	Ganzhou (°C)	Chongqing (°C)
1	9.5	4.9	10.3	8.1	8.2	7.9
2	11.3	7.2	12.3	9.9	9.8	10
3	14.5	11.2	15.3	13.3	13.6	13.8
4	17.9	17.4	20.7	19	19.6	18.5
5	20.1	22.4	24.6	23.3	23.8	22.6
6	21.3	25.8	27.3	26.4	27.1	25.1
7	21	29.2	29	28.2	29.4	28.3
8	20.7	28.3	28.7	28.2	28.8	28.3
9	19.4	23.9	26.4	25.6	25.8	24.1
10	17	18.4	22.5	21.1	21.2	18.6
11	12.9	12.8	17	15.8	15.4	14.2
12	9.6	7.3	11.9	10.6	10.3	9.3

Table S2. Average rainfall (mm) ofCitrus sinensis collection site from January to December that recorded from 1981 to 2010 for Huaning, Huaihua, Shaoguan, Guilin, Ganzhou and Chongqing.

Month	Place					
	Huaning (mm)	Huaihua (mm)	Shaoguan (mm)	Guilin (mm)	Ganzhou (mm)	Chongqing (mm)
1	16.6	73.7	69.3	65.7	65.1	19.7
2	17.6	94.6	106	99.2	113.1	23.3
3	18.1	139.6	177.9	136.9	181.2	43.2
4	34.8	187.9	221.1	217.8	195.4	95.2
5	91.1	181.1	248.9	324.5	233.3	145.9
6	140.5	223.6	249.6	395.2	180.2	192.6
7	180.5	142.6	159.4	232.2	115.8	186
8	177.7	107.1	129.1	147.4	134.6	137.9
9	96.9	76.5	100.8	82.2	90.3	105.8
10	75.1	72.6	43.8	66.8	66	85.8
11	44.9	81.4	51.9	73.1	48	48.3
12	15.1	47.4	41.7	46.8	38.2	24.3

Table S3. Average sunshine duration hours (h) ofCitrus sinensis collection site from January to December that recorded from 1981 to 2010 for Huaning, Huaihua, Shaoguan, Guilin, Ganzhou and Chongqing.

Month	Place					
	Huaning (h)	Huaihua (h)	Shaoguan (h)	Guilin (h)	Ganzhou (h)	Chongqing (h)
1	224.5	76.2	89.7	66.9	87.8	20.6
2	219.6	63	67.5	50	73.5	29.7
3	255.4	69.4	57.6	51.6	72.3	64.9
4	244.8	88.3	75.5	72.6	100.5	93.6
5	212.2	122.8	113.9	109.5	137.8	109.4
6	135	144.8	150.5	131.1	173.1	97.7
7	124.3	238.3	226.6	199.4	261	158.6
8	144.9	229.6	206.7	204.1	235.8	167
9	123.5	160	177.6	193.4	182.1	106.6
10	143.7	133.4	164	157.1	165.2	50.4
11	169.8	115.7	147	134.4	147.1	35.9
12	200	103.2	140.8	117.2	142.1	20.4
